# Enhanced NO_2_ Sensing Performance of Graphene with Thermally Induced Defects

**DOI:** 10.3390/ma14092347

**Published:** 2021-04-30

**Authors:** Namsoo Lim, Hyeonghun Kim, Yusin Pak, Young Tae Byun

**Affiliations:** 1 Sensor System Research Center, Korea Institute of Science and Technology (KIST), Seoul 02792, Korea; namsoo@kist.re.kr (N.L.); yusinpak@kist.re.kr (Y.P.); 2 School of Engineering Technology, Purdue University, West Lafayette, IN 47907, USA; kim3589@purdue.edu

**Keywords:** graphene, defects, rapid thermal annealing, nitrogen dioxide, gas sensor

## Abstract

This paper demonstrates the enhanced NO_2_ sensing performance of graphene with defects generated by rapid thermal annealing (RTA). A high temperature of RTA (300–700 °C) was applied to graphene under an argon atmosphere to form defects on sp^2^ carbon lattices. The density of defects proportionally increased with increasing the RTA temperature. Raman scattering results confirmed significant changes in sp^2^ bonding. After 700 °C RTA, I_D_/I_G_, I_2D_/I_G_, and FWHM (full width at half maximum)(G) values, which are used to indirectly investigate carbon-carbon bonds’ chemical and physical properties, were markedly changed compared to the pristine graphene. Further evidence of the thermally-induced defects on graphene was found via electrical resistance measurements. The electrical resistance of the RTA-treated graphene linearly increased with increasing RTA temperature. Meanwhile, the NO_2_ response of graphene sensors increased from 0 to 500 °C and reached maximum (R = ~24%) at 500 °C. Then, the response rather decreased at 700 °C (R = ~14%). The results imply that rich defects formed at above a critical temperature (~500 °C) may damage electrical paths of sp^2^ chains and thus deteriorate NO_2_ response. Compared to the existing functionalization process, the RTA treatment is very facile and allows precise control of the NO_2_ sensing characteristics, contributing to manufacturing commercial low-cost, high-performance, integrated sensors.

## 1. Introduction

Since the first demonstration of two-dimensional (2D) graphene devices by Andre Geim and Konstantin Novoselov [1], graphene-related researches have increased explosively owing to the excellent electrical [2,3,4], thermal [5], mechanical [6], and chemical [7] properties of graphene. In particular, graphene is considered a very promising gas sensing material. As a representative 2D material, graphene has a high surface-to-volume ratio (~2600 m^2^/g), facilitating the adsorption of gas molecules [8,9]. Furthermore, its low thermal-noise property improves its detection limit [10], and ultra-high carrier mobility (200,000 cm^2^/Vs) [2] and thermal conductivity (5000 Wm^−1^ K^1^) [5] can guarantee rapid transport of electrical signal induced by gas adsorption.

Based on the material properties as mentioned above, various graphene sensors for the detection of O_2_ [11], CO_2_ [12], NH_3_ [13,14], H_2_S [15], et cetera [8] have been studied, demonstrating competitive gas sensing performance compared to existing metal oxide gas sensors. In most studies till now, enhanced gas responsivity and selectivity were associated with covalent functionalization [7], electrical field effect [10,13], and heterojunction with nanomaterials [8,15]. However, these enhancement techniques are not practical due to unstable adsorption of functional or decoration materials on graphene and complex fabrication of sensor structures (e.g., three-terminal electrodes).

Recently, facile methods based on the formation of lattice defects (mostly vacancies) on graphene sensing channels were studied to improve the sensing performance [9,16]. Lee et al. reported that lattice defects could be readily formed on graphene physically via reactive ion etching (RIE), and their sensors showed remarkable responsivity enhancement in NO_2_ and NH_3_ detection. Furthermore, they demonstrated that the defect sites could be energetically more favorable for functionalization or decoration with nanomaterials. Although the RIE technique is very facile and appropriate for mass production, the accumulated damage to carbon-carbon bonds and increased oxidation sites after ion bombardment process severely affected charge transport property, and as such, the responsivity was decreased.

This report successfully demonstrates a facile method of creating graphene defects via rapid thermal annealing (RTA) to improve the performance of NO_2_ gas sensors. The generated graphene defects were confirmed by Raman spectra before and after RTA treatment. The RTA-treated graphene showed a remarkable increase in electrical resistance at metal-graphene-metal (MGM) contacts. Meanwhile, the NO_2_ response of graphene sensors exhibited the maximum responsivity of 24% and the response time of 111 s after the RTA treatment at 500 °C, which is about a two-fold enhancement compared with pristine (not RTA treated) graphene sensor (14%/304 s). Our results suggest that the RTA treatment can be a breakthrough approach to improving the NO_2_ sensing characteristics at low cost, thus contributing to manufacturing commercial low-cost, high-performance, integrated sensors.

## 2. Materials and Methods

### 2.1. Generation of Graphene Defects by RTA Process

The purchased graphene on SiO_2_/Si substrate (Graphene Square Inc., Suwon, Korea) was used without further treatment, and the customized RTA system (see supplementary Figure S1) was utilized to generate graphene defects. The graphene sample was placed in the RTA chamber under an argon atmosphere. Then, the temperature of the chamber was rapidly increased to a target value in 20 s. Three RTA temperatures (300, 500, and 700 °C) were applied. After maintaining the heating state for an hour, the chamber naturally cooled down to room temperature.

### 2.2. Raman Spectra Analysis

Raman spectra were recorded using an InVia Raman microscope (Renishaw Ltd., Gloucestershire, UK) and Nd:YAG laser (λ = 532 nm) at room temperature. The laser power was kept below 1 mW and the grating was 2400 L/mm. Wire program (version 3.4) was utilized to precisely designate peak position and intensity.

### 2.3. Fabrication of Graphene Gas Sensors

A shadow mask was used in DC magnetron sputtering process (SC-701MK II Advance, Sanyu Electron Co., Tokyo, Japan) to construct interdigitated gold (Au) electrodes. During the sputtering, ~315 V_DC_ was applied under an argon atmosphere (Pressure: ~7.0 Pa). The sputtering continued for 7 min with a deposition rate of ~0.5 nm/s. The spacing and the thickness of the electrodes were 100 μm and 200 nm, respectively.

### 2.4. Nitrogen Dioxide (NO_2_) Sensing Measurements

The NO_2_ responsivity of the graphene sensor was analyzed in the custom-built measurement system (the temperature of the sample stage was maintained at 150 °C to improve the sensor’s recovery performance). At first, dry air as a balance gas was injected at 1 V_DC_ and maintained for 20 min to eliminate unwanted gases and water molecules in the chamber. Then, the NO_2_ valve was opened, and the total flow, including the balance and target gases, was 500 sccm. The change in electrical resistance in response to the NO_2_ adsorption was recorded through electrical feed-throughs connecting to a SourceMeter (Model 2400, Keithley Instrument Inc., Cleveland, OH, USA) and sent to Lab-view software via a GPIB-to-USB converter for data collection and analysis.

## 3. Results and Discussion

**Figure 1:**
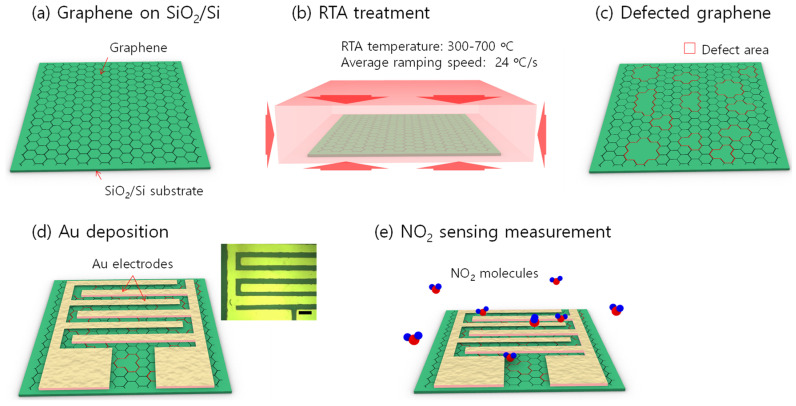
The schematic fabrication process of the chemiresistive-type gas sensor using defected graphene; (**a**) Preparation of a graphene-transferred SiO_2_/Si substrate. (**b**) Rapid thermal annealing (RTA) treatment to form defects in graphene lattice. (**c**) Schematic image of the defected graphene. (**d**) Au electrode deposition by sputtering process with a shadow mask (inset shows an optical microscope image of the device (scale bar: 200 μm)). (**e**) NO_2_ gas sensing measurement in a custom-built gas sensing system.

**Figure 2:**
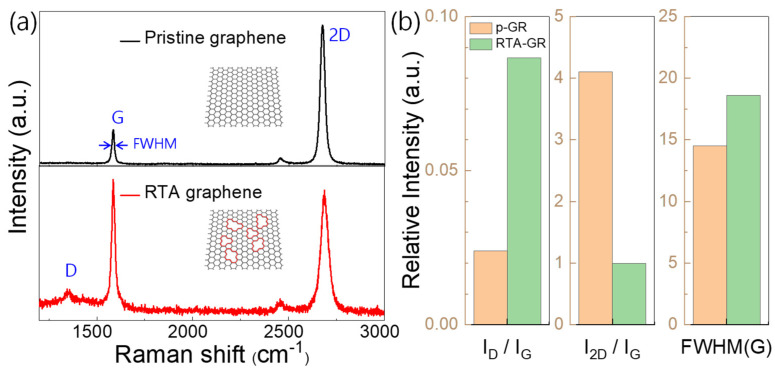
Comparison of Raman spectra of pristine- and RTA-treated graphenes; (**a**) Raman spectra of pristine- (upper) and RTA-treated (lower) graphenes (each inset shows schematic sample image). (**b**) Comparison of I_D_/I_G_, I_2D_/I_G_, and FWHM(G) values of pristine- and RTA-treated graphenes which are extracted from the Raman spectra.

A schematic fabrication process of the defected graphene-based gas sensor is shown in Figure 1. The purchased graphene on SiO_2_/Si substrate was used without further treatment (a), and the rapid thermal annealing (RTA) process was utilized to generate defects in graphene lattices (b). Previously, the RTA process was reported as a tool for generating defects on single-walled carbon nanotube (SWCNT) walls [17,18,19]. Therefore, we expected the same effect of RTA (i.e., generating defects) on graphene whose lattice structure is the same as the SWCNTs. The graphene defects were generated by increasing the RTA temperature rapidly to the target value under an inert argon atmosphere. We tested three different RTA temperatures, namely, 300, 500, and 700 °C. Here, the average ramping speed was ~24 °C/s. The RTA-generated graphene defects were confirmed by Raman spectra in comparison with those of pristine graphene (c) (the details are discussed in Figure 2). We also confirmed the graphene defects indirectly by a transmission electron microscope (TEM) image of SWCNT defects generated at the same RTA condition (see Figure S2).

Subsequently, Au-electrodes were sputtered through a shadow mask to fabricate the metal-graphene-metal (MGM) structured device (d). Au is generally used as electrode material in graphene devices because of its low contact resistance with graphene [20]. The inset shows an optical image of the fabricated device. Finally, the NO_2_ sensing performance of the fabricated device was measured in a custom-built measurement system (e). The used measurement system is further discussed in Figure S3.

Figure 2a shows the Raman spectra of pristine graphene and 700 °C RTA-treated graphene, respectively (insets show schematic sample images). In both cases, graphene lattices were identified by two characteristic peaks (G peaks and 2D peaks at 1590 cm^−1^ and 2700 cm^−1^, respectively). Particularly in the case of pristine graphene (upper), D peak (at 1350 cm^−1^) is very weak and the I_2D_/I_G_ value is relatively high as ~4, demonstrating a defect-free graphene monolayer [21] (here, I_2D_ and I_G_ refer to the intensities of 2D peaks and G peaks, respectively). On the other hand, the RTA-treated graphene shows an increased D peak intensity with a decreased I_2D_/I_G_ value as ~1 in its Raman spectrum (lower), demonstrating the RTA-generated defects. Furthermore, three typical parameters extracted from each Raman spectrum are compared regarding the graphene qualities in Figure 2b. The I_D_/I_G_ value increased from 0.024 to 0.065, meaning increased defect density after RTA treatment. Furthermore, the I_2D_/I_G_ value decreased from 4.1 to 1.0, and the FWHM(G) value increased from 14.5 to 18.6 (here, FWHM refers to the full width at half maximum). These parameters are generally utilized for evaluating the graphene quality, and all the parameter value changes demonstrated the degradation of graphene quality, implying the increased defect density of graphene [22,23,24]. 

**Figure 3:**
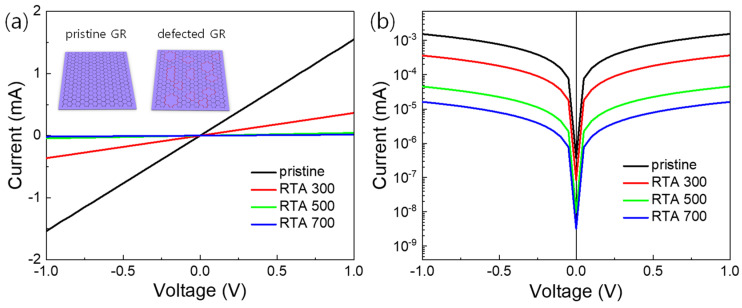
Comparison of the four current-voltage (I–V) curves of graphene-based MGM devices in (**a**) linear- and (**b**) logarithmic scales (The devices were fabricated with pristine graphene and RTA-treated graphenes at 300, 500, and 700 °C, respectively).

Figure 3a shows a comparison of the current-voltage (I–V) curves of the MGM devices fabricated with pristine graphene and RTA-treated graphene samples at 300, 500, and 700 °C (insets show the schematic sample images). As the RTA temperature increases, the current level of the corresponding device gradually decreases. The result suggests that defects are generated in graphene lattices by a high-temperature RTA treatment, and the defect density increases with increasing the RTA temperature. The variation of current levels depending on RTA temperature is similar to those in previous studies using SWCNTs (a carbon allotrope with the same sp^2^ lattices) [17,18,19], suggesting that defect-formation by RTA treatment is also applicable to graphene. Figure 3b shows the I–V curves on a logarithmic scale. The curves are symmetrical with respect to the line ‘V = 0’. The I–V curves’ linearity and symmetry demonstrate the ohmic contact between graphene and the sputtered Au electrodes [25].

**Figure 4:**
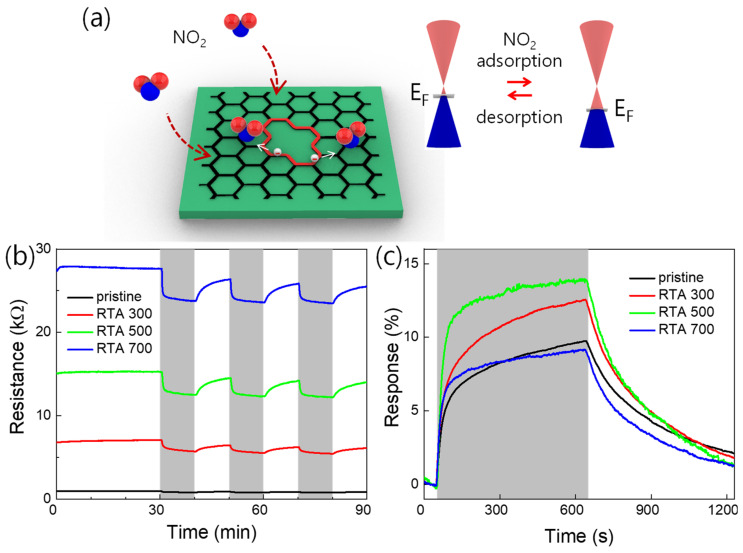
(**a**) Schematic illustration of the expected NO_2_ gas-sensing mechanism of the defected graphene. Comparisons of the time-dependent (**b**) resistance- and (**c**) response curves of the four MGM devices (pristine graphene and RTA-treated graphenes at 300, 500, and 700 °C, respectively).

Additionally, NO_2_ gas sensing performance of the fabricated device was studied. The expected NO_2_ sensing mechanism of the defected graphene device is described in Figure 4a. In general, graphene behaves as p-type material in the air by the following reaction [20,21].

$$O_{2}+2H_{2}O+4e^{-}\to 4{\mathrm{OH}}^{-}$$

when NO_2_ molecules adsorb onto the graphene surface, the concentration of major carriers (i.e., holes) increases by the following reaction [26].

$${\mathrm{NO}}_{2}\to {\mathrm{NO}}_{2}{}^{-}+h^{+}$$

Meanwhile, the RTA-generated defect regions (highlighted by a red line) are locally electron-abundant due to the presence of oxygen-containing functional groups (e.g., epoxy-, carbonyl-, and ether groups) [16]. Thus, adsorption of the electron-withdrawing NO_2_ molecules is promoted, which enhances the NO_2_ response. The resistance change can be explained by the Fermi level shift (see the inset of Figure 4a). Here, the oxygen-containing functional groups are expected to generate when the defected graphene is exposed to air.

Figure 4b shows a comparison of time-dependent resistance curves measured with four different graphene-based NO_2_ sensors (pristine graphene and RTA-treated graphenes at 300, 500, and 700 °C, respectively). The gray areas indicate where the target gas is injected. Here, the target- and base gases are 5 ppm NO_2_ (balanced by N_2_) and dry air, respectively, and 1 V_DC_ was applied for each measurement. Additionally, the temperature of the sample stage was maintained at 150 °C during the measurement to improve the recovery performance. As the RTA temperature increased, the resistance of the corresponding device also increased. This is because the defect density increases as the temperature increases, as discussed above. However, in all the cases, the resistance decreased when the device was exposed to NO_2_ gas, meaning that all the device channels are p-type without polarity change.

The time-dependent response curves are compared in Figure 4c. The response is defined by the following formula.

$$Response\;\left(R,\;\%\right)=\frac{\left(R_{g}-R_{0}\right)\times 100}{R_{0}}$$

Here, *R_g_* and *R*_0_ are the minima- and maxima resistance values, respectively, in the area where target gas is injected. As the RTA temperature increases from 0 to 500 °C, the response of the corresponding device increases. However, the response somewhat decreases in the case of the RTA-treated sample at 700 °C. This phenomenon can be understood as the defect density increased as the RTA temperature increased, and the lattice structures were significantly damaged by high thermal energy at above a critical RTA temperature, resulting in deterioration of NO_2_ response.

**Figure 5:**
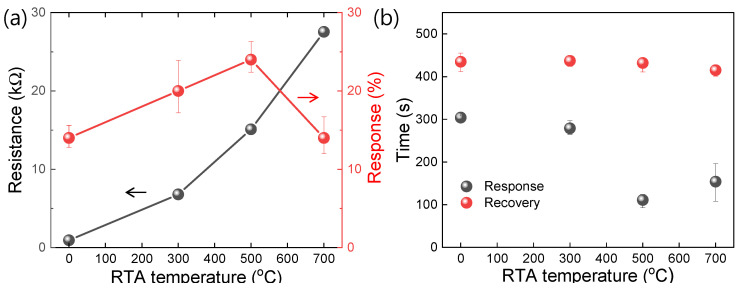
(**a**) Variation of resistance and response values depending on the RTA temperature. (**b**) Comparison of the response- and recovery times of the devices depending on the RTA temperature.

In Figure 5a, the resistance and response values are quantitatively compared depending on RTA temperature. Note that the resistance and response refer to each response curve’s initial resistance value and the average of three response values, respectively, in Figure 4b. As the RTA temperature increased, the resistance of the corresponding graphene device also increased. Interestingly, when the RTA temperature increased to 500 °C, the response increased to ~24%. However, the response decreased to ~14% in the case of RTA treatment at 700 °C. As discussed above, this result can be understood as the defect density increasing as the RTA temperature increases. However, the lattice structure partially collapsed, and the gas response decreased above a critical RTA temperature. The result suggests that if a defected graphene prepared under the optimal RTA condition is used for a sensing material, the off current (I_off_) can be reduced, and the response can be enhanced as well.

Additionally, each reaction curve’s response- and recovery times are compared (Figure 5b). The response (or recovery) time is defined as the elapsed time to reach 90% (or 10%) of the maximum response value from the turn-on (or turn-off) time. Interestingly, the response time tends to decrease as the RTA temperature increases (304 s for the 0 °C RTA sample and 154 s for the 700 °C RTA sample). The calculation process is discussed more in Figure S4. This phenomenon can be understood as a fact that the adsorption of electron-withdrawing NO_2_ gas molecules is promoted by the electron-abundant defected area as discussed above. However, the recovery times are almost the same for all the samples (~430 s), meaning that the RTA-generated defects do not affect the desorption rate of NO_2_ molecules.

## 4. Conclusions

In this paper, we demonstrated that graphene defects could be generated by RTA processes, and they could be utilized in high-performance NO_2_ sensors. As the RTA temperature increased from 300 to 700 °C, the corresponding device’s resistance gradually increased, whereas the NO_2_ response increased to 500 °C and decreased at 700 °C. This is because the defect density increased as the RTA temperature increased, and the graphene lattice is highly damaged above a critical RTA temperature, resulting in the deterioration of NO_2_ response. Interestingly, as the RTA temperature increased, the corresponding sensor’s response time decreased. The result implies that adsorption of NO_2_ molecules is promoted by the electron-abundant graphene defect areas. Our results suggest that the RTA process can be utilized to generate tunable graphene defects, and the defected graphene can be applied to high-performance gas sensor studies.

## Data Availability

All data has been included in the paper.

## Supplementary Materials

The following are available online at https://www.mdpi.com/article/10.3390/ma14092347/s1, Figure S1: The customized RTA system; photographs of (a) front and (b) back sides of the equipment, (c) a picture inside the RTA chamber (inset shows a schematic structure). Figure S2: TEM image of the RTA-generated defected SWCNTs. Figure S3: The customized gas sensing measurement system; (a) a photograph of the system, (b) a schematic image of the system, pictures (c) inside the gas chamber and (d) of the gas chamber during measurement. Figure S4: Comparison of response times of the defected graphene-based NO_2_ sensors (each figure shows the typical response curve of the NO_2_ sensor prepared with RTA-treated graphene); (a) pristine graphene, (b) 300 °C -, (c) 500 °C -, and (d) 700 °C RTA graphenes.
