# Supplementary material for: Enhanced NO2 Sensing Performance of Graphene with Thermally Induced Defects
Source: Materials (Basel). 2021 Apr 30;14(9):2347. doi: 10.3390/ma14092347 (PMC8124493; doi:10.3390/ma14092347)
Supplement: Supplementary file 1 [file materials-14-02347-s001.zip › materials-1198861-supplementary.pdf]

materials

MDPI

Article

Enhanced NO_2 Sensing Performance of Graphene with Thermally Induced Defects

Namsoo Lim [1], Hyeonghun Kim [2], Yusin Pak [1] and Young Tae Byun [1,*]

[1] Sensor System Research Center, Korea Institute of Science and Technology (KIST), Seoul 02792, Korea; namsoo@kist.re.kr (N.L.); yusinpak@kist.re.kr (Y.P.)
[2] School of Engineering Technology, Purdue University, West Lafayette, IN 47907, USA; kim3589@purdue.edu
* Correspondence: byt427@kist.re.kr

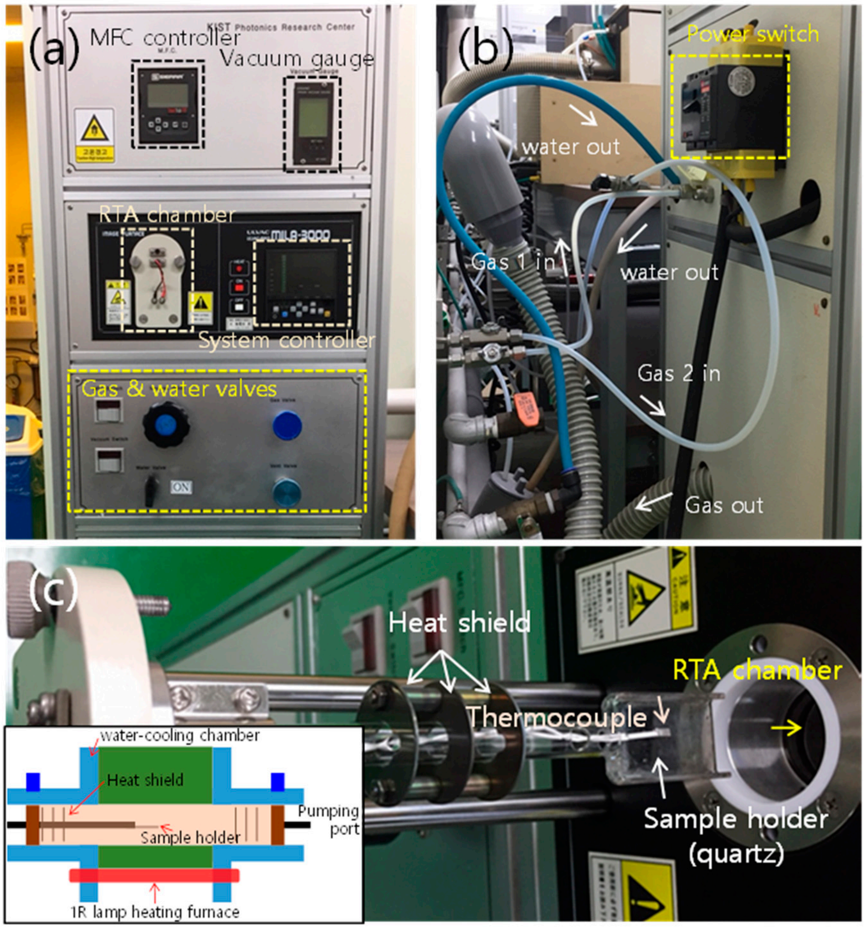

Figure S1. The customized RTA system; photographs of (**a**) front and (**b**) back sides of the equipment, (**c**) a picture inside the RTA chamber (inset shows a schematic structure).

Citation: Lim, N.; Kim, H.; Pak,Y.; Byun, Y.T. Enhanced NO_2 Sensing Performance of Graphene with Ther-mally Induced Defects. *Materials* **2021**, *14*, 2347. https://doi.org/10.3390/ma14092347

Academic Editor: Sergei Kulinich

Received: 11 April 2021
Accepted: 29 April 2021
Published: 30 April 2021

Publisher's Note: MDPI stays neutral with regard to jurisdictional claims in published maps and institutional affiliations.

Copyright: © 2020 by the authors. Submitted for possible open access publication under the terms and conditions of the Creative Commons Attribution (CC BY) license (http://creativecommons.org/licenses/by/4.0/).

Figures S1a and b show the front and the back sides of the customized RTA system used in this paper. The RTA parameters (*i.e.*, gas flow rates, water flow, vacuum level of the chamber) are tuned by various switches on the front panel, and the gases and water are fed into the chamber through the lines indicated by arrows. Here, two gas lines were connected to argon (for RTA) and N_2 (for venting), respectively. Figure S1c shows a picture inside the RTA chamber. The temperature of the chamber can rise rapidly *via* optical heating method using 1R lamp.

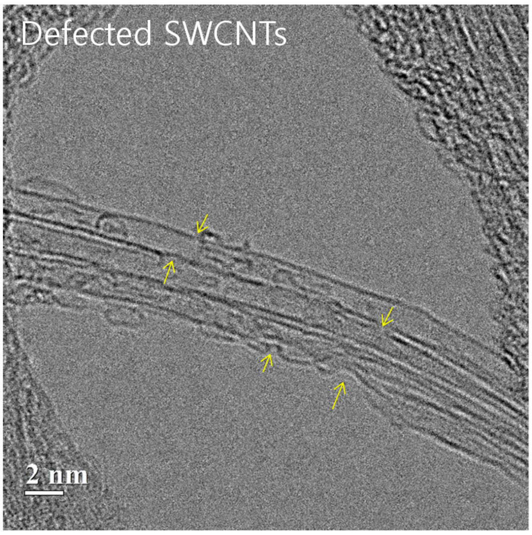

Figure S2. TEM image of the RTA-generated defected SWCNTs.

Figure S2 shows the defected SWCNTs which was RTA-treated at 500 °C. The size of defects in sp^2 carbon lattice (yellow arrows) is less than 2 nm. Because graphene has the same sp^2 lattice with SWCNTs', the same RTA effect (*i.e.*, generating lattice defects) is expected for graphene.

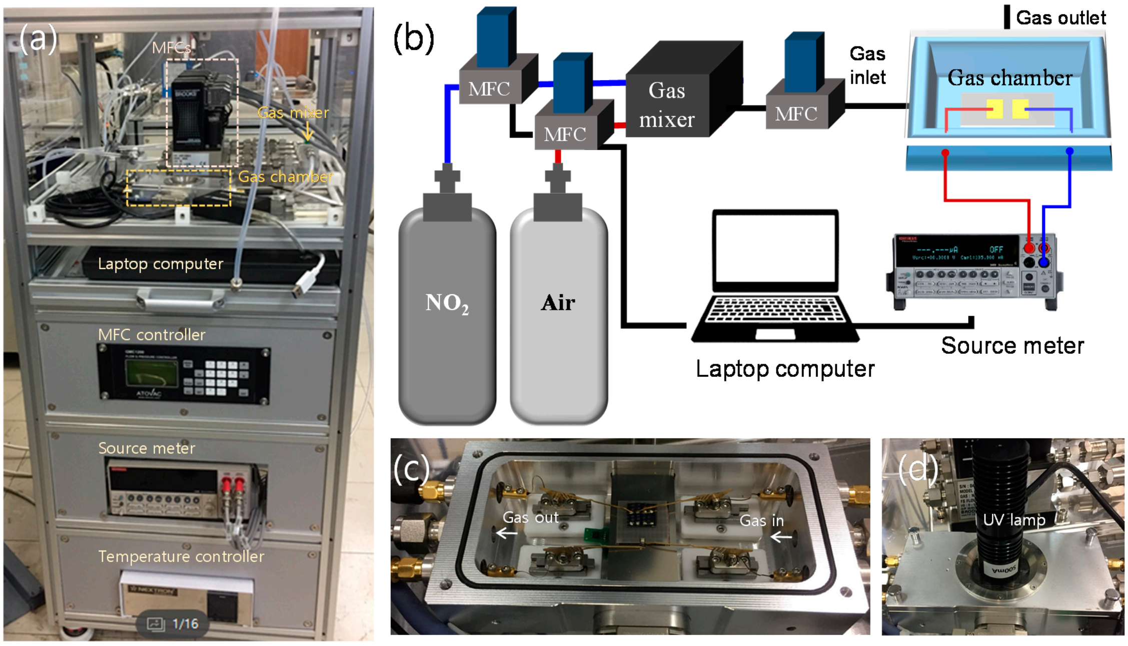

Figure S3. The customized gas sensing measurement system; (a) a photograph of the system, (b) a schematic image of the system, pictures (c) inside the gas chamber and (d) of the gas chamber during measurement.

Figures S3a and b show a photograph and a schematic image of the customized gas sensing measurement system used in this paper. All the parameters (*i.e.*, gas flow rate, bias voltage, temperature) were controlled and monitored using the labtop computer. Figure S3c shows inside the gas chamber in which a device is connected. During the measurement, the sensor was heated or UV-irradiated for fast recovery (Figure S3d).

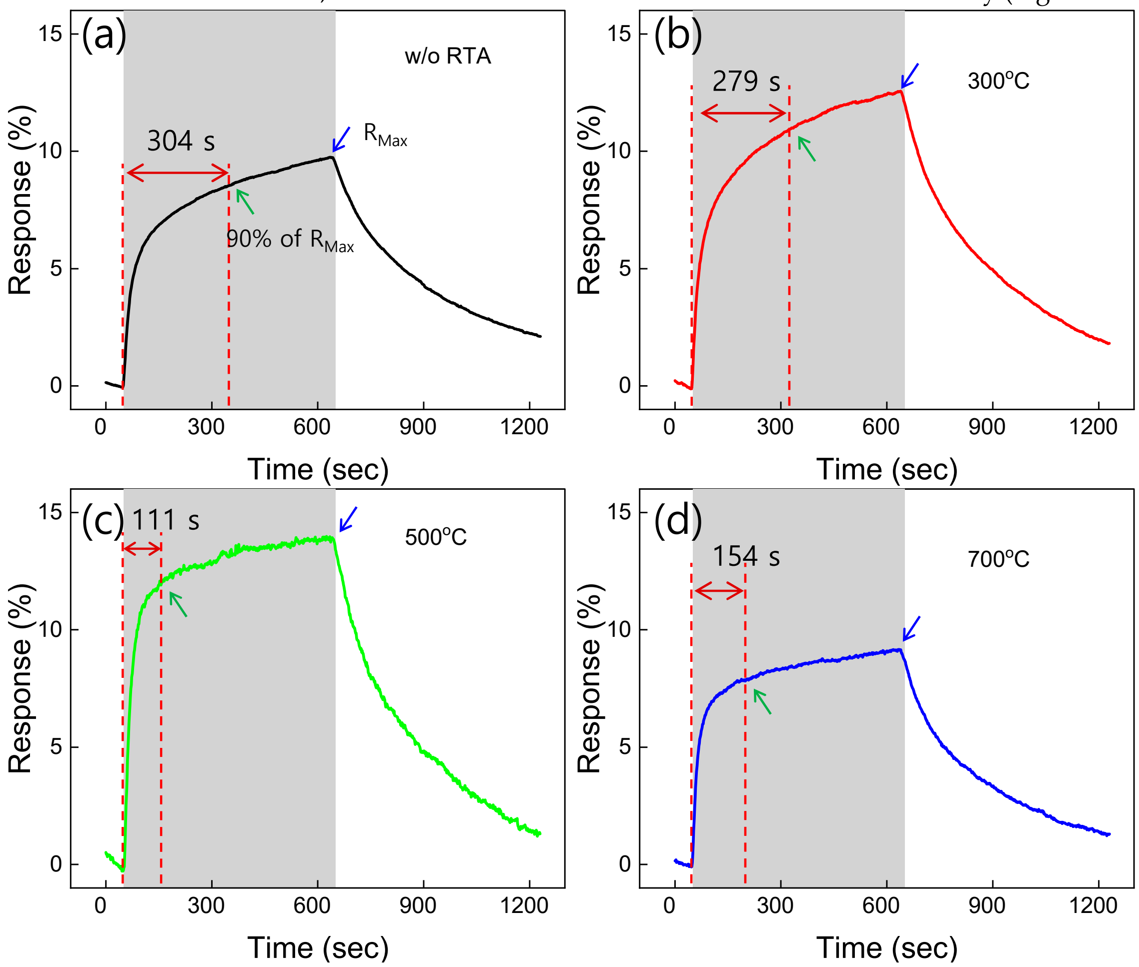

Figure S4. Comparison of response times of the defected graphene-based NO_2 sensors (each figure shows the typical response curve of the NO_2 sensor prepared with RTA-treated graphene); (a) pristine graphene, (b) 300 °C-, (c) 500 °C-, and (d) 700 °C RTA graphenes.
